# Case Report: Pansclerotic Morphea-Clinical Features, Differential Diagnoses and Modern Treatment Concepts

**DOI:** 10.3389/fimmu.2021.656407

**Published:** 2021-03-09

**Authors:** Sarah Ventéjou, Agnes Schwieger-Briel, Rebecca Nicolai, Stephanie Christen-Zaech, Caroline Schnider, Michael Hofer, Sofia Bogiatzi, Daniel Hohl, Fabrizio De Benedetti, Marie-Anne Morren

**Affiliations:** ^1^Pediatric Dermatology Unit, Department of Pediatrics and Dermatology and Venereology, University Hospital Lausanne and University of Lausanne, Lausanne, Switzerland; ^2^Department of Dermatology, Pediatric Skin Center, University Children's Hospital Zurich, Zurich, Switzerland; ^3^Division of Rheumatology, Istituto di Ricovero e Cura a Carattera Scientifico, Ospedale Pediatrico Bambino Gesù, Rome, Italy; ^4^Department of Pediatric Rheumatology, University Hospital Lausanne and University of Lausanne, Lausanne, Switzerland; ^5^Laboratory of Dermato-Histopathology, Department of Dermato-Venereology, University Hospital Lausanne and University of Lausanne, Lausanne, Switzerland

**Keywords:** pansclerotic morphea, stiff skin, scleroderma, tocilizumab, IL-6, case report

## Abstract

Pansclerotic morphea (PSM) is a rare skin disease characterized by progressive stiffening of the skin with or without the typical superficial skin changes usually seen in morphea (localized scleroderma). Standard therapy, consisting of a combination of systemic glucocorticoids and methotrexate or mycophenolate mofetil, does rarely stop disease progression, which may lead to severe cutaneous sclerosis and secondary contractures. Little is known about the efficacy of newer biologicals such as abatacept, a fusion protein antibody against CTLA-4, or tocilizumab, a fully humanized IL-6R antibody, in the treatment of this pathology. We present the case of an 8 years old girl with an unusual, progressive stiffening of the skin, which was eventually diagnosed as pansclerotic morphea. A treatment with systemic glucocorticoids and methotrexate combined with tocilizumab led to a good clinical response within 2 months after initiation. In this paper, we discuss differential diagnoses to be considered and this new promising treatment option based on a case review of the literature.

## Introduction

Diseases associated with stiff skin are extremely rare in children (1). The differential diagnoses include a generalized presentation of localized scleroderma (especially the deep variant of pansclerotic morphea (PSM)], systemic sclerosis, scleroderma-like disorders such as scleredema and eosinophilic fasciitis, or stiff skin disease. Differentiating these disorders is not always straightforward.

In this case report we describe an 8 years old child, presenting with a slowly progressing skin thightness after an initial infiltrated plaque at the anterior neck. Laboratory values, lung function tests, capillaroscopy, cardiac and ophthalmologic investigations were largely unremarkable. Skin MRI showed oedema of muscle fasciae. The final diagnosis was pansclerotic morphea, based on course and histopathology. We highlight the diagnostic dilemma and show the excellent response to a treatment combining systemic glucocorticoids, methotrexate and tocilizumab.

## Case Report

**Figure 1:**
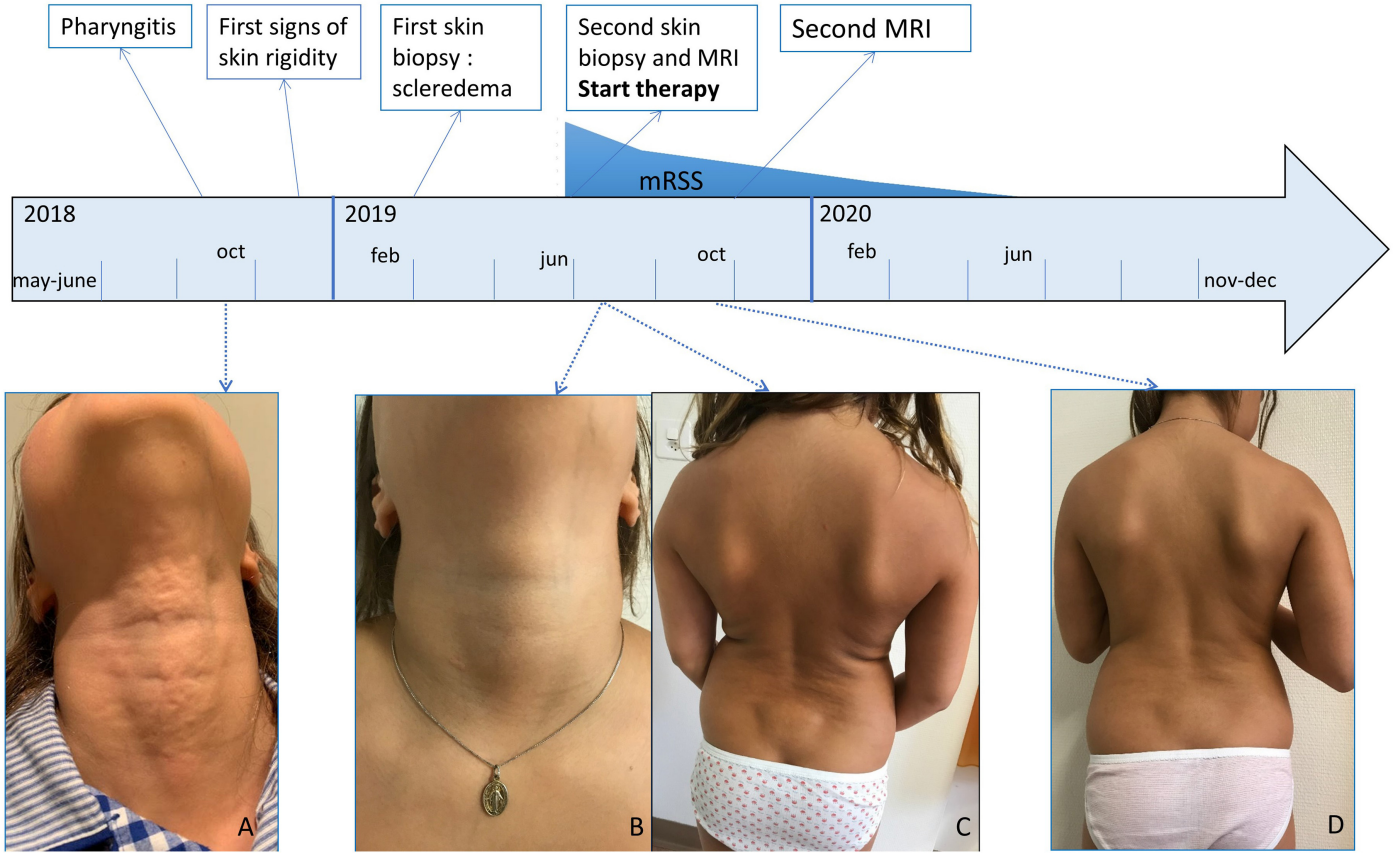
Clinical presentation. **(A)** Irregular plaque at the anterior neck, **(B)** lesion neck healed, **(C)** wooden hard skin, most severe at the hips and lower back, only sparing face, hands, feet and neck, **(D)** after 4 months of treatment with systemic glucocorticoids, methotrexate and tocilizumab softening of the skin. mRSS, modified Rodnan Skin Score.

An 8-year-old girl in good general health, presented with an irregular infiltrated plaque on the anterior neck of 3 months duration (Figure 1A). The family reported a streptococcal throat infection treated with antibiotics 1 week prior to onset. Antistreptolysin antibodies were high at that time. A skin biopsy, showed mucin accumulation between thickened collagen fibers suggestive of scleredema Buschke.

However, over the following months, the child developed a slowly progressive stiffness and reduced mobility of the back, shoulders and hips, subsequently extending to the wrists, fingers and knee with restriction of the flexibility. Raynaud phenomenon was absent.

After seven months, she presented at our consultation with a generalized wooden hard, infiltrated skin, most prevalent at the posterior thighs and lower back, only sparing her face, feet, hands and fingers [modified Rodnan Skin Score (mRSS) = 29/51]. The original neck lesion had disappeared (Figures 1B,C). Skin was adherent to the underlying tissues on palpation, with some nodular infiltrations and impossible to fold. Her general health remained well. She was able to continue school activities, tennis and artistic gymnastics although she felt more and more restrained. There were no episodes of fever or other systemic complaints. Rheumatologic investigation showed restricted movements of the wrists and knees, without clear signs of arthritis, therefore thought to be secondary to the tight skin.

**Figure 2:**
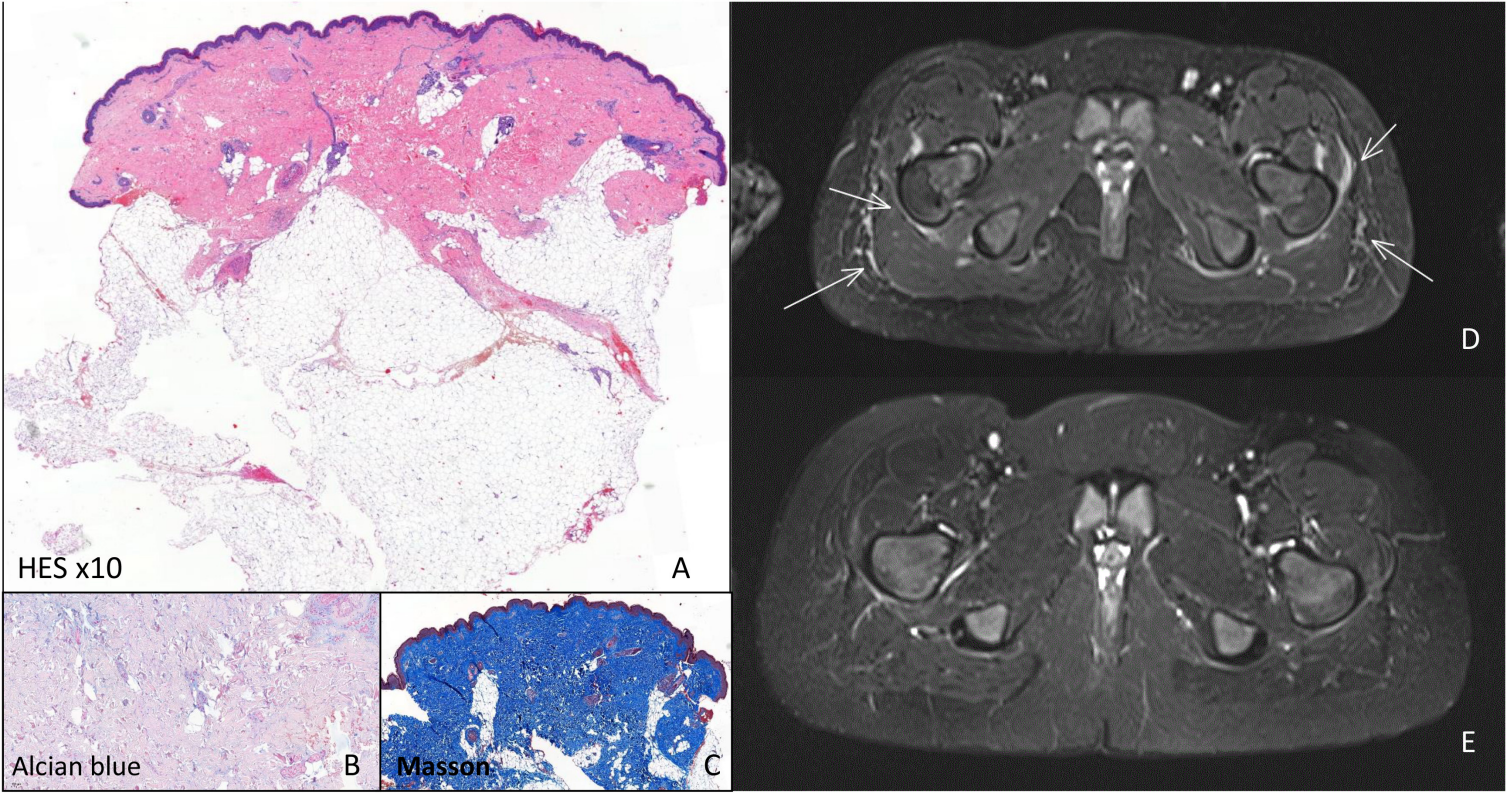
Histology and MRI investigations. Histology of indurated skin lesion before start of systemic treatment: **(A)** HE stain: Enlarged dermis with coarse collagen fibers invading part of the hypodermis, underneath the sweat glands, with a sparse perivacular lymphohistiocytic infiltrate, **(B)** only minimal mucin detected by alcian blue stain, **(C)** Masson stain confirmed the presence of coarse collagen (blue). MRI investigations. **(D)** Before start of systemic therapy showing oedema of the muscular fasciae (arrow), **(E)** 4 months after the start of systemic treatment oedema has disappeared.

At that time a deep skin biopsy of the thigh showed an enlarged dermis with a sparse perivascular lymphohistiocytic infiltrate, without eosinophils, and coarse hypertrophic collagen fibers. They invaded part of the hypodermis, underneath the sweat glands, surrounding fat globuli, causing compression on skin adnexes but did not invade deeper layers. A Masson stain confirmed the presence of coarse collagen but only minimal mucin was present (Figures 2A–C).

Blood-work was unremarkable with only at the time of the second biopsy minimally elevated eosinophils, slight hypergammaglobulinemia, but negative auto-antibodies (rheumatoid factor, ANA esp. anti-dsDNA, Scl70, RNP, SS-A, SS-B, SmD-1, Jo-1, anti-β2GP1, anti-cardiolipin, lupus anticoagulant) and normal kidney function, liver and muscle enzymes. On whole body MRI widespread oedema of fascia and subcutis was present (Figure 2D).

Lung function tests, capillaroscopy, cardiac and ophthalmologic investigations were within normal range. This together with absence of acral sclerosis and Raynaud phenomenon makes a diagnosis of systemic sclerosis highly improbable.

Due to this severe clinical status, suspecting PSM, 7 months after the first symptoms, an aggressive combined treatment with glucocorticosteroid pulses (methylprednisolone 30 mg/kg/day for 3 days) followed by prednisone 2 mg/kg/day orally on a tapering regimen over 6 months, methotrexate 16.5 mg/m^2^/week and tocilizumab 10 mg/kg intravenously followed by weekly subcutaneous injections of 4.5 mg/kg/week were started. Our patient was encouraged to continue her sports activities, instead of adding physiotherapy, to stimulate her mobility skills.

Already after 1 month, a slight improvement was noted, which became more obvious after 4 months (Figure 1D). The skin felt less tight and hardened (mRSS = 18/51). With the exception of hyperpigmentation of a skin region where a band aid was placed, there was no alteration in skin coloration. Gymnastic skills improved drastically and this was confirmed by clinical examination showing less restriction in wrists and knees mobility as well as by disappearance of oedema on MRI (Figure 2E). Further improvement occurred the following months when she remained under methotrexate and tocilizumab treatment alone (mRSS = 6/51). The hyperpigmented patch disappeared slowly. Skin returned to normal, 1 year after start of treatment (mRSS = 0/51). Further lowering of therapy will be considered after 1 year, if no relapse occurs.

## Discussion

Stiffening of the skin associated with systemic symptoms, Raynaud phenomenon, characteristic lesions on capillaroscopy, and the presence of auto-antibodies esp. ANA, are diagnostic for systemic sclerosis, which is an extremely rare disease in childhood.

When systemic complaints and Raynaud phenomenon are absent, pansclerotic morphea (PSM), scleroderma-like disorders especially scleredema and eosinophilic fasciitis or stiff skin disease have to be considered (Table 1) (1). At onset, as our case illustrates, patients may be difficult to categorize, as there is a lack of clear diagnostic criteria, of disease markers and pathophysiology is not well-known, as isn't the relationship between theses entities. Illustrating this, there are many case reports showing clinically and/or histologically overlapping features of different entities (2–6).

**Table 1:** Typical clinical characteristics of PSM, eosinophilic fasciitis, scleredema and stiff skin syndrome.

	**Pansclerotic morphea (PSM)**	**Eosinophilic fasciitis**	**Scleredema**	**Stiff skin syndrome**
Age at onset	Infants to teenagers rare in adults	Infants to adults	Child (post-infectious), adults	Toddler to infant, M: 1,6 a; max: 7a (sporadic or familial)
Location at onset	All body areas possible	Extremities Rapid extension within weeks	Back of the neck Extension in few days but can vary (2–8 w)	Pelvic and shoulder girdles Minimal progression
Final distribution	Diffuse, circumferential, centrifugal extension; sparing hands and feet, progressive	Diffuse; sparing face and trunk	Face, head and neck, trunk and shoulders; sparing extremities spontaneous resolution within months (exc. 2 years)	Diffuse
Skin (characteristic changes)	Hard to touch, hypo- or hyper-pigmentations, atrophic sclerotic plaques Ulcers	Painful induration of the skin Peau d'orange, Erythema or pigmentation Prayer's & groove sign*	Induration of the skin, Pigmentation, Peau d'orange	Rock-hard skin bound to underlying Tissues, Hypertrichosis, Hyperpigmentation
Articulations	Restricted joint mobility, contractures	Restricted joint mobility, contractures	Restricted joint mobility	Restricted joint mobility, contractures
Systemics symptoms Or visceral involvement	Asthenia *Secondary*: compartment syndrome, dysphagia, dyspnea	Exceptional	Exceptional (cardiac)	Not reported
Capillaroscopy	Normal	Normal	Normal	Not reported
Biology	ANA in 30% of cases; eosinophilia and hypergammaglobuline-mia: inconstant	ANA negative; eosinophilia and hypergammaglobulinemia (may be absent) ± increased aldolase	normal (ASLO +)	Normal
Histology	Epidermis: thinned Dermis/hypodermis (may extend into fascia): inflammation and thickened collagen bundles Perivascular inflammation: lymphocytes and plasma cells. Eosinophils may be present	Epidermis: normal Dermis: normal Hypodermis and fascia: edema, inflammation and thickened collagen bundles Inflammation: lymphocytes, eosinophils, histiocytes and plasma cells	Epidermis: normal Dermis: thickened x3- 4, full of collagen fibers Containing clear spaces full of mucin ++ Hypodermis: invaded by collagen fibers Fascia: respected No inflammation	Epidermis: normal Dermis: increased mucin and fibroblast, horizontal collagen fibers Hypodermis: adipocytes entrapment Fascia: thickened and hyalinized No inflammation
IRM	Inconstant œdema of hypodermis and fascia	Oedema of hypodermis and fascia	Not reported	Not reported

* *Prayer sign: when the patient is unable to oppose the palmar surfaces of both hands with extended wrists. Groove sign: depression along the course of the superficial veins. M, median*.

The clinic and histopathology of the first lesion in the neck (although not in the typical posterior location), preceded by a streptococcal infection, were suggestive for scleredema (1) in this particular case. In children this disorder is usually caused by an infection, runs a very acute course and can lead to skin stiffness, which tends to involve preferentially the back neck and shoulder girdle. On histology, the disease is characterized by mucin accumulation between coarse collagen fibers. Spontaneous resolution is the rule within months. However, in our case, the development of a severe involvement of the pelvic girdle, protracted course and the findings of the second biopsy proved this first diagnosis wrong.

Stiff skin disease usually is most marked at the pelvic girdle as in our patient. However it is a monogenic disease, caused by an heterozygous mutation in the fibrillin-1 gene (*FBN1*), with onset always before the age of 6 years in the diffuse form. Moreover, this disease tends to remain stable or is only minimally progressive over the years (7, 8).

Eosinophilic fasciitis has a subacute onset and tends to affect extremities, trunk and neck but spares hands and feet (1, 2). Histopathology shows inflammation and fibrosis mainly at the fasciae and in the lower subcutis (6). However, in our case, although fascia was affected on MRI, on histology deep subcutis and fascia were spared. Moreover, skin and blood eosinophilia were absent in most analyses. Response to corticosteroids, is usually excellent, however, relapse after stopping is the rule.

Therefore, the most likely diagnosis in this patient was PSM. This disease, a rare (<1%) subtype of localized scleroderma (9), is characterized by near total body surface involvement, with circumferential lesions, sparing fingers and toes that usually extend in subcutaneous tissue, and may affect fascia, muscle and bone. It has a more insidious onset than eosinophilic fasciitis and post-infectious scleredema, symptoms progressively appearing over months (10). Atrophic hyperpigmented lesions and joint contractures (therefore called disabling PSM) are usually present but may also develop later in the course of disease. On histopathology fibrosis and inflammatory infiltrates always involve lower dermis and upper subcutis, sometimes deeper tissues may also be affected (6). In about one third of patients, auto-antibodies are present and sometimes eosinophilia and hypergammaglobulinemia can be seen.

Much progress has been made in clarifying the role of profibrotic cytokines such as TGFβ, IL-4 and IL-6 [for a review see (11)] in scleroderma pathogenesis. More specifically, IL-6 is a pro-inflammatory cytokine produced by B-cells, T-cells, monocytes and fibroblasts. It is required for differentiation of Th17-cells. IL-6 also regulates fibroblasts activity, stimulates collagen production and inhibits the synthesis of collagenases. In systemic sclerosis it has been demonstrated that IL-6 blockade reverses TGFβ activation (12). Via these actions, IL-6 has a role in the fibrotic pathways (11, 13). High serum levels of IL-6 have been described in patients with localized scleroderma (13–17).

According to literature, treatment of PSM is disappointing (18). Disease progression, despite classical treatments (systemic glucocorticoids, methotrexate or mofetil mycophenolate) is often reported. New treatments targeting pathways stimulating collagen production such as IL-6 (tocilizumab), or broader T-cell activation (abatacept) have been evaluated. Until now, four reports, on tocilizumab treatment of seven longlasting pediatric PSM cases, not responding to standard treatments, have been published. There was a partial response (especially on activity scores), in 6 out of 7 patients (18–21), within months. Details about these and our patients are given in Table 2. Positive experiences with abatacept are especially reported in patients with systemic sclerosis and different types of morphea. However, there are no patients with PSM treated at this moment as far as we know (22, 23).

**Table 2:** Pediatric case reports of PSM treated with tocilizumab.

**References**	**Age at onset (years)**	**Previous treatments**	**Disease duration at onset of TCZ**	**Dose of tocilizumab**	**Concomitant treatment**	**Result**
Martini et al. (19) Patient 1	4	Prednisolone (pulse and oral) MTX 15 mg/m^2^/w MMF 700 mg/m^2^/d Imatinib 200 mg/d	+/−144 m	IV 8 mg/kg/8 w	Still on Imatinib? None?	Rapid reduction of inflammation follow-up (FU) 18 m
Patient 2	4	Prednisolone (pulse and oral) MTX 10–15 mg/m^2^/w MMF 700 mg/m^2^/d	+/− 68 m	IV 8 mg/kg/8 w	Prednisone + MTX + MMF	Inactivation of lesions allowing reduction and stop of TCZ, FU of 24 m after stop still inactive
Foeldvari et al. (20) Patient 1	7	MTX 15 mg/m^2^/week MMF 1,200 mg/day	81 m	IV 8 mg/kg every 2 weeks	Prednisone + Tacrolimus	No new lesions; decrease in erythema and skin thickness after 31 m
Patient 2	2	MTX 15 mg/m^2^/week MMF 40 mg/kg/day Etanercept 0.8 mg/kg/week	95m	IV 8 mg/kg every 4 weeks	MMF	No new lesions, decrease of erythema, skin thickness stable after 23 m
Patient 3	7	MTX 24 mg/m^2^/w	9 m	SC 6.5 mg/kg every 3 weeks	MTX	No new lesions, decrease of skin thickness after 9 m
Zang et al. (21)	6	Prednisolone pulses MTX MMF	+/−42 m	IV 300 mg/4 w	MTX	Improvement within months After 18 m mLoSSI *improved from 22 to 6 PGA-A** improved from 30 to 7
Soh et al. (18)	4,5	Naproxen, prednisolone (pulses and oral) MTX (15 mg/m^2^/w) MMF 300 mg BID IVIG Rituximab	+/− 96 m	dose not specified	Prednisone + MTX + MMF + Ig IV Hydroxychloroquine and naproxen	After 3 months no effect
This case report	9	none	7 m	IV 10 mg/kg followed by SC 4.5 mg/kg/w	Prednisolone pulse and oral MTX 16,5 mg/m^2^/w	Improvement of skin infiltration started after 1 m and was marked after 4 m of treatment, skin returned to normal after 12 m: mRSS from 29 to 0/51

* mLoSSI modified Localized Scleroderma Skin Severity Index.

** *PGA-A Physician Global Assessment of Disease Activity score. mRSS, modified Rodnan Skin Score*.

In our case the combination of systemic glucocorticoids, methotrexate and tocilizumab led to a rapid, complete and sustained healing. The specific role of tocilizumab is difficult to evaluate, because all three medications were started simultaneously. However, considering the disappointing results of standard treatments, it seems likely that it played a major role in reversing the fibrosis in our patient. Furthermore, the response in this case suggests that early treatment with tocilizumab is even more effective, leading to complete healing.

In conclusion, with our case, we discuss the differential diagnoses of stiff skin in children and describe some potential difficulties in making an early diagnosis of PSM. This case also suggests that drugs targeting IL-6 might be a good option to consider early in the treatment of PSM, when it might be most beneficial, in this disease where standard treatments usually are disappointing.

## Data Availability Statement

The original contributions generated for this study are included in the article/supplementary material, further inquiries can be directed to the corresponding author/s.

## Ethics Statement

Written informed consent was obtained from the minor(s)' legal guardian/next of kin for the publication of any potentially identifiable images or data included in this article.

## Author Contributions

AS-B was the first physician to see the patient. SV, M-AM, RN, CS, MH, and FD followed the patient clinically. SB and DH reviewed the histopathology. SV and M-AM wrote the first draft of the article. All authors contributed to and agreed the final draft.

## Conflict of Interest

The authors declare that the research was conducted in the absence of any commercial or financial relationships that could be construed as a potential conflict of interest.

## References

[1] Ferreli C Gasparini G Parodi A Cozzani E Rongioletti F Atzori L. Cutaneous manifestations of scleroderma and scleroderma-like disorders: a comprehensive review. Clin Rev Allerg Immunol. (2017) 53:306–36. 10.1007/s12016-017-8625-4 28712039

[2] Odhav A Hoeltzel MF Conty K. Pansclerotic morphea with features of eosinophilic fasciitis: distinct entities or part of a continuum? Ped Dermatol. (2014) 31:e42–7. 10.1111/pde12279 24383741

[3] Farrington ML Haas JE Nazar-Stewart V Mellins ED. Eosinophilic fasciitis in children frequently progress to scleroderma like fibrosis. J Rheumatol. (1993) 20:128–32. 8441144

[4] Azevedo VF Serafini SZ Werner B Müller CS Franchini CF Morais RL. Stiff skin syndrome versus scleredema: a report of two cases. Clin Rheumatol. (2009) 28:1107–011. 10.1007/s10067-009-1178-z 19415378

[5] Huemer M Seeber A Huemer C. Scleroderma-like syndrome in a child: eosinophilic fasciitis or scleredema adultorum? Eur J Pediatr. (2000) 159:520–2. 10.1007/s004310051323 10923227

[6] Doyle JA Connolly SM Winkelmann RK. Cutaneous and subcutaneous inflammatory sclerosis syndromes. Arch Dermatol. (1982) 118:886–90. 10.1001/archderm.11811886 7138043

[7] Loeys BL Gerber EE Riegert-Johnson D Iqbal S Whiteman P McConnell P. Mutations in fibrillin-1 cause congenital scleroderma: stiff skin syndrome. Sci Transl Med. (2010) 2:23ra20. 10.1126/scitranslmed3000488 20375004 PMC2953713

[8] Myers KL Mir A Schaffer JV Meehan SA Orlow SJ Binster NK. Segmental stiff skin syndrome (SSS): a distinct clinical entity. J Am Acad Dermatol. (2016) 75:163–8. 10.1016/j.jaad.201601038 26944597

[9] Zulian F Arthreya BH Laxer R Nelson AM Feitosa de Oliveira SK Punaro MG. Juvenile localized scleroderma: clinical and epidemiological features in 750 children. Int Study Rheumatol. (2006) 45:614–20. 10.1093/rheumatology/kei251 16368732

[10] Kim A Marinkovich N Vasquez R Jacobe H. Clinical features of patients with morphea and the pansclerotic subtype: a cross-sectional study from the morphea in adults and children cohort. J Rheumatol. (2014) 41:106–12. 10.3899/jrheum130029 24293577 PMC5607739

[11] Torok K Li SC Jakobe HN Taber SF Stevens AM Zulian F. Immunopathogenesis of pediatric localized scleroderma. Front Immunol. (2019) 10:908. 10.3389/fimmu201900908 31114575 PMC6503092

[12] Denton CP Ong VH Xu S Chen-Harris H Modrusan Z Lafyatis R. Therapeutic interleukin-6 blockade reverses transforming growth factor-beta pathway activation in dermal fibroblasts. insights from the fascinate clinical trial in systemic sclerosis. Ann Rheum Dis. (2018) 77:1362–71. 10.1136/annrheumdis-2018-213031 29853453 PMC6104680

[13] Kurzinski KS Torok KS. Cytokine profiles in localized scleroderma and relationship to clinical features. Cytokine. (2011) 55:157–64. 10.1016/j.cyto.201104001 21536453 PMC3632442

[14] Ihn H Sato S Fujimoto M Kikuchi K Takehara K. Demonstration of interleu-kin-2, interleukin-4 and interleukin-6 in sera from patients with localized scleroderma. Arch Dermatol Res. (1995) 287:193–7. 10.1007/BF01262331 7763091

[15] Cox LA Webster GF Piera-Velazquez S Jimenez SA. Multiplex assessment of serum cytokine and chemokine levels in idiopathic morphea and vitamin K 1-induced morphea. Clin Rheumatol. (2017) 36:1173–8. 10.1007/s10067-017-3580-2 28220270

[16] Budzyńska-Włodarczyk J Michalska-Jakubus MM Kowal M Krasowska D. Evaluation of serum concentrations of the selected cytokines in patients with localized scleroderma. Postepi Dermatol Alergol. (2016) 33:47–51. 10.5114/pdia201548044 26985179 PMC4793054

[17] Alecu M Geleriu L Coman G Gălătescu L. The interleukin-1, interleukin-2, interleukin-6 and tumour necrosis factor alpha serological levels in localised and systemic sclerosis. Rom J Intern Med. (1998) 36:251–9. 10822522

[18] Soh HJ Samuel C Heaton V Renton WD Cox A Munro J. Challenges in the diagnosis and treatment of disabling pansclerotic morphea of childhood: case-based review. Rheumatol Int. (2019) 39:933–41. 10.1007/s00296-019-04269-w 30838436

[19] Martini G Campus S Raffeiner B Boscarol G Meneghel A Zulian F. Tocilizumab in two children with pansclerotic morphea: a hopeful therapy for refractory cases? Clin Exp Rheumatol. (2017) 35:211–3. 28980909

[20] Foeldvari I Anton Lopez J Friswell M Bica B de Inocencio J Aquilani A. Tocilizumab is a promising treatment option for therapy resistant juvenile localized scleroderma. J Scleroderma Relat Disord. (2017) 2:203–7. 10.5301/jsrd.5000259

[21] Zang A Nocton J Chiu Y. A case of pansclerotic morphea treated with tocilizumab. JAMA Dermatol. (2019) 155:388–9. 10.1001/jamadermatol.2018.5040 30649148

[22] Castellví I Elhai M Bruni C Airò P Jordan S Beretta L. Safety and effectiveness of abatacept in systemic sclerosis: the EUSTAR experience. Semin Arthritis Rheum. (2020) 50:1489–93. 10.1016/j.semarthrit.2019.12.004 32165035

[23] Li SC Torok KS Ishaq SS Buckley M Edelheit B Ede KC. Preliminary evidence on abatacept safety and efficacy in refractory juvenile localized scleroderma. Rheumatology. (2020) keaa873. 10.1093/rheumatology/keaa873. [Epub ahead of print]. 33369667

